# The Influence of Shc Proteins on the Whole Body Energetic Response to Calorie Restriction Initiated in 3-Month-Old Mice

**DOI:** 10.1155/2014/562075

**Published:** 2014-02-17

**Authors:** Jennifer H. Stern, Kyoungmi Kim, Jon J. Ramsey

**Affiliations:** ^1^VM Molecular Biosciences, University of California, Davis, CA 95616, USA; ^2^Cancer Center Division, University of Arizona, Tucson, AZ 85724, USA; ^3^Department of Public Health Sciences, University of California, Davis, CA 95616, USA

## Abstract

There is increasing evidence that Shc proteins play a role in energy metabolism, and we have previously reported that knockdown of Shc proteins influences the energetic response to acute (3 days) calorie restriction (CR) in 18-month-old mice. Whether Shc proteins play a role in the metabolic response to CR in younger mice has yet to be elucidated. Hence, we sought to determine the impact of 3 days and longer term (2 months) CR on energy expenditure (EE) and respiratory quotient (RQ) in 3 month-old Shc knockout (ShcKO) and wild-type (WT) mice. ShcKO mice decreased (*P* < 0.001) EE normalized for body weight (EE_BW_) by 3 days of CR, while no such change was observed in WT animals. However, both ShcKO and WT mice decreased (*P* < 0.001) EE_BW_ at 2 months of CR and there were no differences in body weight between the ShcKO and WT mice at either 3 days or 2 months of CR. Consistent with increased fatty acid oxidation, only ShcKO mice maintained decreased (*P* < 0.001) 24 h RQ through 2 months of CR, suggesting that they were able to maintain increased fatty acid oxidation for a longer period of time than WT mice. These results indicate that Shc proteins may contribute to some of the acute energetic responses to CR.

## 1. Introduction

Shc proteins act as adaptor molecules which specifically bind to phosphorylated tyrosine residues on the cytoplasmic motif of growth factor receptors, thereby serving as mitogenic signaling molecules. Three splice variants (p46Shc, p52Shc, and p66Shc) are encoded by the mammalian Shc locus. The p66Shc(−/−) mouse has been a common model used to investigate the possible role p66Shc plays in energy metabolism and aging. However, it has recently been shown [1] that the levels of both the p52Shc and p46Shc isoforms are also substantially decreased in liver and skeletal muscle from these animals. Thus, these mice (we refer to as ShcKO) provide a model of overall decreases in Shc protein levels in muscle, liver, and other tissues. While it has been proposed that the p66Shc isoform may play a role in the aging process [2], it is also well established that Shc proteins play a role in insulin signaling [3, 4]. Furthermore, it has been proposed that Shc may influence aging through alterations in insulin signaling, adiposity, and energy metabolism [1, 5]. Still, the overall impact of Shc proteins on energy metabolism is not entirely clear. Previous overfeeding studies in young (2-3 months) mice demonstrated that ShcKO may mitigate weight gain in response to overnutrition. It has been reported that body weights are lower in ShcKO compared to wild-type (WT) mice when consuming either a standard or high fat diet despite the fact that energy intake is not different between genotypes [5]. Additionally, deletion of Shc in leptin-deficient Ob/Ob mice leads to a decrease in weight gain without altering food intake [6]. While there is accumulating evidence that the Shc proteins may play a role in the metabolic response to overnutrition, until our previous study in 18-month-old mice [7], the role of this protein in the metabolic response to calorie restriction (CR) had not been studied.

We previously reported that 18-month-old ShcKO mice do not demonstrate a difference in the energetic response to 2 months of calorie restriction (CR). However, significant differences in whole body energy expenditure after 3 days of CR were observed in ShcKO compared to that of WT mice [7]. It remains to be determined if this effect of Shc proteins on the energetic response to CR is influenced by age, or if a similar response would also be observed in younger ShcKO mice. Hence, the purpose of this study was to determine if Shc proteins contribute to the metabolic response to CR in 3-month-old mice. In the current study, we investigated the impact of ad libitum feeding (baseline), 3 days of CR, and 2 months of CR on EE and RQ in 3-month-old ShcKO and WT mice. These CR time points were selected to represent a period of very active weight loss (3-day-CR) and a period when major weight loss had ceased (2-month CR).

## 2. Materials and Methods

### 2.1. Animals and Diet

All mice in this study were on a C57BL/6J background and have been previously described [1, 2]. Heterozygous crosses were used to generate the founder lines of ShcKO and WT animals. Animals were randomly assigned to one of the following groups:

 Control (WT, *n* = 9)-baseline measures
 Control (ShcKO, *n* = 9)-baseline measures
 CR (WT, *n*  =  9)-baseline, 3-day and 2-month measures
 CR (ShcKO, *n*  =  9)-baseline, 3-day and 2-month measures

All wild-type (WT) and ShcKO mice were 3-4 months of age at the start of the study. The control groups were used for cross-sectional studies investigating EE adjusted for fat free mass and organ mass at baseline in ShcKO compared to WT animals. The control groups were also used for body composition comparisons with the CR groups. Because collection of body composition and organ mass data required termination of the animals, separate control and CR groups were needed. The CR treatment groups were used for longitudinal studies to compare changes in EE, RQ, and BW from baseline to 3 days and 2 months of CR treatment. Prior to collection of indirect respiration calorimetry data, food intake and body weight were monitored for 7 days. Animals were conventionally housed in individual cages in a light (12-h light/12-h dark cycle) and temperature (22°C) controlled vivarium at the University of California, Davis (UCD). All mice were fed a commercial diet (7012 Teklad LM-485 Mouse/Rat Sterilizable Diet, Harlan, USA). Sentinel mice were housed on the same rack as the study mice and exposed to bedding from the cages of the study animals. Two sentinel mice were euthanized every three months for serology. All tests from these mice were negative. This study was approved by the UCD Animal Care and Use Committee.

### 2.2. Food Intake

Ad libitum fed animals had continuous access to food. Ad libitum food intake was measured by weighing the amount of food remaining in the hopper at the same time daily, while accounting for any food spillage found in cages by removing bedding and weighing remaining food particles at the bottom of the cage.

### 2.3. Indirect Respiration Calorimetry

Total daily EE was measured using whole-body indirect respiration calorimetry. Prior to calorimetry measurements, all animals were adapted to the chambers for a period of 24 h at which time food intake was monitored to ensure that these values did not differ from previously collected data during adaptation to individual housing. The ad libitum fed control animals underwent one 24 h data collection period. In the CR treatment groups, mice were housed in the calorimetry chambers from baseline through day 3 of CR. Calorimetry measurements were completed for each animal at baseline (ad libitum fed) and day 3 of CR feeding. Calorimetry measurements were completed again at 2 months after the initiation of CR and immediately following a 24 h adaptation period. Chambers had the same dimensions and shape as the animals' home cage (Paige Instruments, Woodland, CA). Room air was drawn through the chambers at 400 mL/min. This flow rate was controlled and measured with a mass flow controller (MFS-5, Sable Systems International, Las Vegas, NV). Samples of room and chamber air were dried by a Peltier condenser (PC-4, Sable Systems) before entering oxygen and CO_2_ analyzers. Oxygen content was measured by a fuel cell oxygen analyzer (FC-10, Sable Systems) and CO_2_ content was measured by an infrared CO_2_ analyzer (CA-10, Sable Systems). Calorimeter calibration was performed daily prior to beginning each 24 h measurement. A 1.9% CO_2_ reference gas, 100% industrial grade nitrogen gas, and dry room air were used to calibrate the CO_2_ and oxygen analyzers. Data from the mass flow controllers and gas analyzers were collected using a data acquisition system (UI2, Sable systems) with a PC using Expedata software (Version 1.3.0.12, Sable Systems). EE was calculated using the following modified Weir equation [8]:

(1) $$\begin{array}{c} \text{EE}\left(\text{kJ}\right)=\left(16.5\;\text{kJ}/\text{L}\times V_{{\text{O}}_{2}}\right)+\left(4.63\;\text{kJ}/\text{L}\times V_{{\text{CO}}_{2}}\right). \end{array}$$

RQ was calculated as the ratio of volume of CO_2_ produced to the volume of O_2_ consumed. A food quotient of 0.87 was calculated from the proportions of protein, fat, and carbohydrates in the diet.

### 2.4. Calorie Restriction

Immediately following baseline indirect respiration calorimetry measurements (ad libitum conditions), mice assigned to the CR treatment group were given 60% of their food intake measured during ad libitum feeding. This acute phase of CR was maintained for three days. Upon completion of day 3 of CR, animals were given 74% of their ad libitum food intake. This feeding protocol was based on that of our previous study in 18-month-old mice [7]. During all CR periods animals were fed daily at 9:00PM.

### 2.5. Organ Weights and Body Composition

Subsequent to 24 h calorimetry data collection, control animals were sacrificed via CO_2_ inhalation and cervical dislocation. Following collection of calorimetry data at the 2-month time point, CR treatment group animals were sacrificed in the same manner. Immediately following euthanasia, organs were weighed and returned to the carcass at which time the carcass was weighed and stored at −20°C until measurement of fat-free body mass by total body water (TBW) analysis, as previously described [7].

### 2.6. Statistical Analyses

Independent *t*-tests were used to determine if EI, BW, EE, and RQ differed between genotypes at baseline ad libitum conditions. EE is expressed as kJ/min/mouse and kJ/min using BW as a covariate in the model. Analyses were performed on 24 h data and subsequently partitioned by light/dark cycle. To investigate possible differences in the energetic response over time to CR (3 days and 2 months), individual trajectories of changes in EE and RQ over time were compared between genotypes by repeated measures analysis of variance (ANOVA) using linear random effects models. Each response level was entered as the dependent variable. Genotype, time, and genotype*time-point interaction terms were entered as independent variables. To account for between subject heterogeneity in the changes of response levels, intercept and time were modeled as random effects. Multiple comparisons were controlled by the Bonferroni correction method where appropriate. For cross-sectional comparisons, differences in organ weights and FFM between controls and 2-month-old CR animals were determined using independent *t*-tests. At the completion of the 2-month CR treatment, EE adjusted for FFM and crude organ weight was compared between ad libitum fed controls and CR treated mice via analysis of covariance (ANCOVA) with FFM and crude organ weight as covariates. To ensure that EE of the cross-sectional and longitudinal baseline groups did not differ, ANOVA was used to compare baseline EE between these two groups. Significance was defined as a two-sided *P* < 0.05. All statistical analyses were performed using SAS version 9.3 (SAS Institute, Inc.).

## 3. Results

### 3.1. Energy Intake

Mean EI in both the CR and control groups during the 24 h baseline/ad libitum calorimetry data collection period did not differ significantly between ShcKO and WT mice (control group baseline: 2.88 g ± 0.13 and 3.08 g ± 0.10, resp., *P* = 0.248; CR group baseline: 2.97 g ± 0.13 and 2.93 g ± 0.20, resp., *P* = 0.875).

### 3.2. Body Weight and Organ Weights

**Figure 1:**
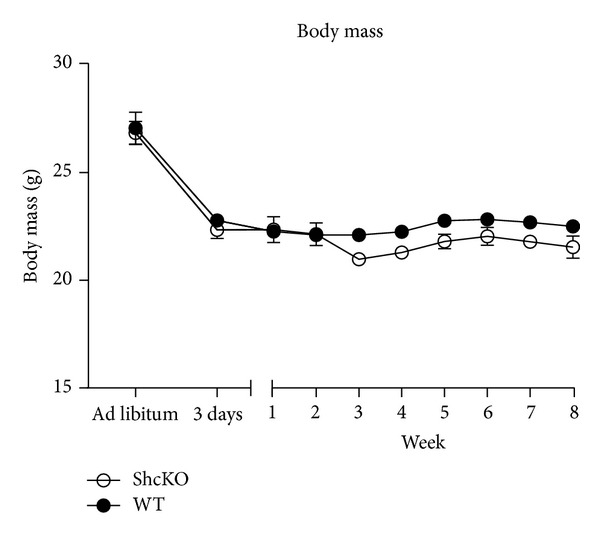
Body weights at baseline, 3-days after initiating CR, and subsequent 8 weeks of CR treatment in ShcKO and WT mice. Data are presented as means ± SEM. * baseline differed from all other time points, ANOVA Bonferroni corrected (*P* ≤ 0.001).

Body weight did not differ between ShcKO and WT mice at baseline (26.83 g ± 0.516 and 27.07 g ± 0.246, resp., *P* = 0.69) (Figure 1). In both genotypes, there was a decrease (*P* < 0.001) in body weight at 3 days and 2 months of CR compared to baseline (Figure 1). Furthermore, the trajectory of change in BW in response to CR did not differ by genotype (Figure 1). However, there was an overall genotype effect with BW being lower in the ShcKO compared to WT mice.

Cross-sectional analyses revealed that the change in organ weights in response to CR did not differ between genotypes (Table 1). Also, the change in crude organ weight (sum of liver, kidneys, heart, and brain) did not differ significantly between ShcKO and WT mice (Table 1). Interestingly, while FFM at the completion of the 2-month CR period did not differ significantly between ShcKO and WT mice, FFM in the ad libitum fed control group was significantly lower in ShcKO compared to that of WT mice (*P* < 0.05) (Table 1).

As expected, when comparing body composition in ad libitum fed control animals to that of mice that underwent 2 months of CR treatment, both ShcKO and WT animals demonstrated significantly reduced BW, FFM, and crude organ weight in CR treated mice compared to ad libitum fed controls. Furthermore, both ShcKO and WT mice demonstrated significant decreases in the weights of liver, kidney, and spleen. The change in organ weight in response to CR was not different between genotypes (*P* < 0.05).

### 3.3. Energy Expenditure

**Figure 2:**
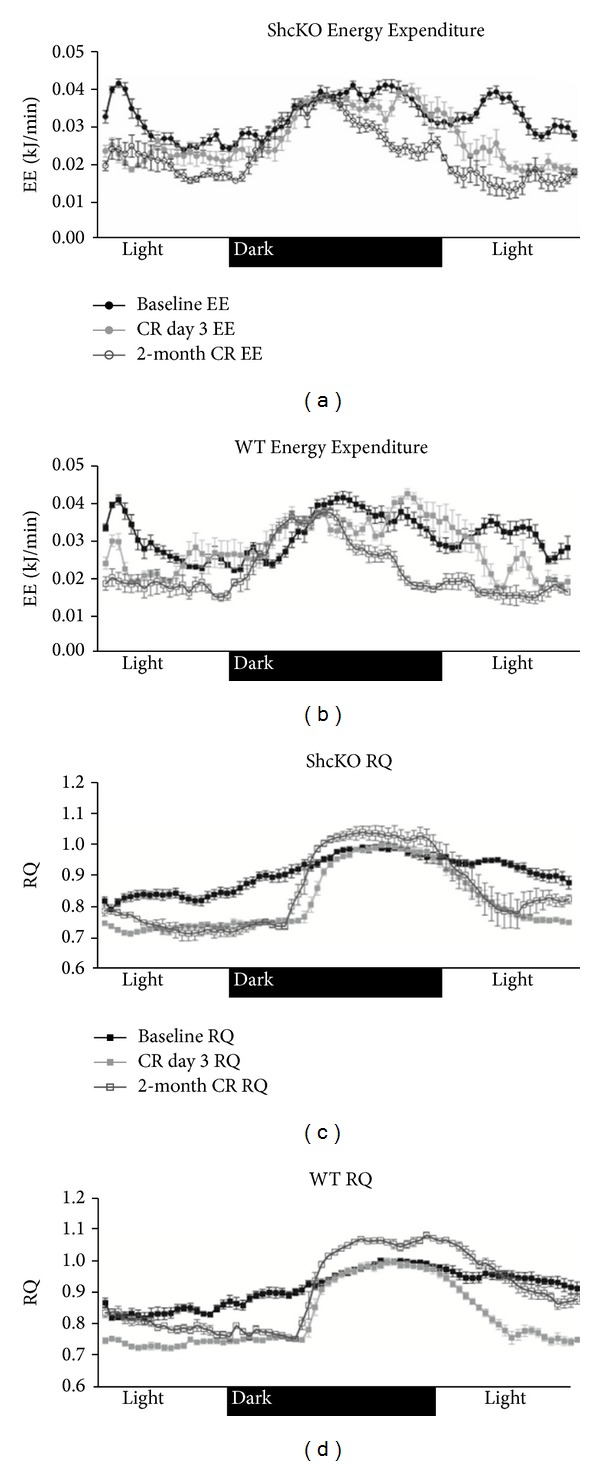
24 h energy expenditure in (a) ShcKO and (b) WT mice and RQ in (c) ShcKO and (d) WT mice under ad libitum, acute, and 2-month CR conditions. Data are presented as means ± SEM.

Longitudinal analyses revealed that, under 24 h ad libitum conditions, average EE did not significantly differ between ShcKO and WT mice, regardless of whether BW was used as a covariate in the model (Table 2). Figures 2(a) and 2(b) show the change in EE over 24 h at baseline, 3-day CR, and 2-month CR time points. The pattern of change in EE normalized for body weight (EE_BW_), however, was different between ShcKO and WT mice. While ShcKO showed a significant decrease (*P* < 0.001) in EE_BW_ by 3 days of CR, WT mice only decreased EE_BW_ by 2 months of CR (Table 3). This data was partitioned into light and dark cycle to determine if specific patterns of diurnal change in EE were responsible for these genotype differences. The decrease in EE at 3 days of CR in the ShcKO mice was entirely due to a decrease in light cycle EE, since dark cycle EE was not significantly altered in these animals. Both the ShcKO and WT animals showed a decrease (*P* < 0.001) in EE_BW_ compared to baseline at 2 months of CR. In the case of the ShcKO mice, the decrease in EE_BW_ compared to baseline and 3 days was due to decreases (*P* < 0.001) in both light and dark cycle EE. In contrast, the decrease in EE_BW_ at 2-month CR compared to baseline and 3-day CR in the WT mice was entirely due to a decrease (*P* < 0.001) in light cycle EE. In the WT mice, there were no significant differences in dark cycle EE_BW_ between baseline, 3-day CR, and 2-month CR (Table 3).

Cross-sectional comparisons were used to investigate whether CR induced changes in EE adjusted for FFM or crude organ mass (ORGAN) differed between ShcKO and WT mice (Table 3). There were no differences (*P* > 0.05) in EE and EE_BW_ (*P* > 0.05) between ShcKO and WT mice in the control groups (baseline measurements only). EE and EE normalized for fat-free body mass (EE_FFM_) or organ mass (EE_ORGAN_) was decreased in both the ShcKO and WT mice at 2 months of CR, and there was no evidence of genotype difference in this CR-induced decrease in EE. The same results were observed when the data was partitioned into 24 h, light or dark cycle EE.

### 3.4. Respiratory Quotient

**Figure 3:**
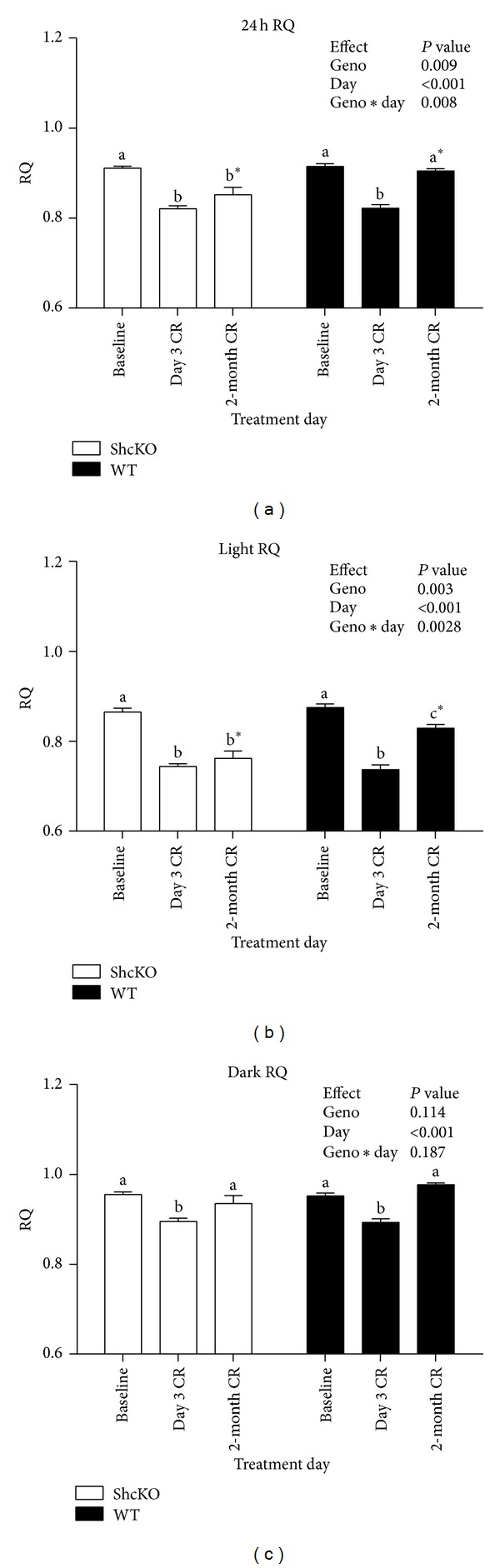
24 h (a), light (b), and dark (c) RQ under baseline, 3 day, and 2-month CR conditions. Data are presented as means ± SEM. Letters that differ indicate difference within genotype, between treatment days, *P* < 0.05; *indicates difference between genotype, within treatment day, *P* < 0.05.

At baseline, 24 h average RQ did not significantly differ between ShcKO and WT mice (Figure 3(a)). However, the trajectory of change in RQ from baseline to 3-day CR to 2-month CR differed between ShcKO mice and WT mice. Both ShcKO and WT mice decreased 24 h RQ significantly in response to 3 days of CR treatment (*P* < 0.001 for both groups). However, while WT animals demonstrated a significant increase in 24 h RQ from day 3 of CR to 2 months of CR treatment, ShcKO animals maintained this CR-induced RQ depression. Figures 2(c) and 2(d) show the distribution of change in RQ over 24 h at baseline, 3-day CR, and 2-month CR treatments. Because animals were fed shortly after the dark cycle began, as expected, there was a significant effect of light/dark cycle on RQ. Based on this result, subsequent analyses were also performed by light and dark cycle separately.

3-day CR induced a decrease in dark cycle RQ in both ShcKO and WT mice when compared to baseline (*P* < 0.001). Dark cycle RQ returned to baseline levels for both ShcKO and WT mice by 2 months of CR (Figure 3(c)). There was no evidence of a genotype difference in this CR-induced RQ decrease.

WT animals showed a decrease (*P* < 0.001) in light cycle RQ at 3 days of CR compared to either baseline or 2-month CR. In the WT mice, light cycle RQ returned to baseline levels by 2 months of CR. In contrast, ShcKO showed a decrease (*P* < 0.001) in light cycle RQ at 3 days compared to baseline, and this difference was maintained through 2 months of CR. This decrease in light cycle RQ is primarily responsible for the difference in 24-hour RQ between genotypes at 2 months of CR. These results indicate that ShcKO mice were able to maintain lipid oxidation for a longer period of time following initiation of CR than the WT animals.

## 4. Discussion

We have previously demonstrated in 18-month-old mice that knocking down Shc proteins may influence the energetic response to 3 days of CR by enhancing the CR-induced decrease in EE. However, we found that it does not impact the whole animal energetic response to 2 months of CR. Because the majority of studies initiate CR at a younger age than 18 months, and many of the studies indicating that Shc proteins may play a role in the metabolic response to overnutrition have been conducted in young animals [1, 5, 6], it was important to investigate the impact of Shc proteins on CR-induced changes in whole body energy metabolism in a younger adult cohort of mice.

CR induces a decrease in EE. In the present study, a decrease in EE was observed by 3 days of CR and continued through 2 months of CR in both the WT and ShcKO mice. However, when BW was used as a covariate, only the ShcKO showed a decrease in EE_BW_ by 3 days of CR while EE_BW_ was only decreased in the WT mice at 2 months of CR. This result is similar to the CR study in 18-month-old ShcKO and WT mice which also found that only the ShcKO mice showed a decrease in EE_BW_ by 3 days of CR [7]. The fact that similar CR-induced changes in EE were observed in both the present study and 18-month-old ShcKO mice supports the idea that the level of Shc proteins influences the initial drop in energy expenditure in response to CR. The mechanism through which Shc proteins may influence energy expenditure is not entirely known. It has been reported that p66Shc localizes to mitochondria and stimulates respiration in mouse embryonic fibroblasts [9]. This finding is consistent with the idea that low Shc levels may promote a decrease in oxygen consumption and EE. It is also important to note that the decrease in 3-day EE_BW_ in the ShcKO mice is due to a decrease in light cycle EE. Light cycle in mice is often a reflection of resting energy expenditure since food intake and physical activity EE occur predominantly at night [10, 11]. Torpor generally occurs in the light cycle and there is some evidence that CR can induce bouts of light cycle torpor in C57Bl/6 mice [11–13]. Thus, it is possible that torpor could be impacting the CR induced changes observed in this study. It has been reported that ShcKO mice show a faster drop in body temperature than WT animals when exposed to cold [5, 14], although it is not known if the ShcKO mice lose body temperature or enter torpor more quickly than WT mice under conditions that do not involve severe cold exposure. Additional studies are needed to determine the influence of Shc proteins on body temperature and torpor in CR mice. Nonetheless, the results of the present study suggest that Shc proteins do influence the initial drop in EE in response to sustained CR.

By 2 months of CR, both the ShcKO and WT mice showed decreases of similar magnitude in EE adjusted for BW, LBM, or organ mass. There are at least two possible explanations to explain why differences in EE response to CR between genotypes were limited to only very acute CR (3 days). First, the magnitude of the decrease in EE_BW_ was relatively small (less than 10%) between the WT and ShcKO mice. It is possible that this change could become lost with additional EE changes in response to sustained CR. Several signaling molecules (AMPK, SIRT1/SIRT3, and PGC-1*α*) appear to play a role in the metabolic/energetic adaptation to CR [15–20], and it may be challenging to separate the effects of Shc proteins from those induced by other signaling molecules with CR. Second, if decreased Shc levels do play a role in the metabolic adaptation to CR, it would be expected that WT animals would also show a drop in Shc levels. If this is the case, ShcKO and WT mice may both show the effects of low Shc levels with sustained CR. Additional work is needed to determine the influence of CR on the levels of Shc proteins.

In contrast to studies that have found differences in body weight change in response to overnutrition between ShcKO and WT mice [5, 6], the present study shows that there is no difference between genotypes in body weight change induced by 3 days or 2 months of calorie restriction initiated in 3-month-old mice. These results are similar to those observed in WT and ShcKO mice started on CR at 18 months of age [7] and indicate that the decrease in EE observed with very acute CR (3 days) in the ShcKO mice was not of sufficient magnitude or duration to induce clear changes in body weight between genotypes. However, it is worth noting that age at which CR was initiated did have a major influence on the magnitude of weight loss in both the WT and ShcKO mice. The magnitude of weight loss following 2 months of CR was much greater in 18-month-old mice [7] compared to the 3-month-old mice used in our present study. While a decrease in body mass is the most obvious physiological response to CR treatment, the degree of this decrease varies greatly among studies and is dependent on the level and duration of the restriction, as well as age, sex, and strain of the animal [21–23]. Body weight curves for ad libitum fed C57BL/6 mice show that peak body mass reached around 18 months of age and is often followed by involuntary weight loss [24]. The energetic stresses placed on mice by CR are different if the animal is a young adult and can divert some energy from tissue growth versus an older animal which may have difficulty maintaining weight. Thus, the results of the present study highlight the fact that the examination of different ages of CR initiation is necessary to truly determine if observed changes in energy metabolism are universal or unique to a particular age group.

In both the ShcKO and WT mice, RQ decreased from baseline to 3 days of CR. However, only the ShcKO mice continued to show depressed RQ values through 2 months of CR. It has previously been reported that the activities of enzymes involved in fatty acid oxidation are increased in fasted ShcKO compared to WT mice [25]. The results of the present study are consistent with the idea that capacity for fatty acid oxidation is increased in the ShcKO versus WT mice. However, these changes in fatty acid oxidation may be age-dependent since no difference in pattern of RQ change was observed between genotypes when CR was started at 18 months of age [7]. The physiological consequences of the sustained decrease in RQ in the 3-month CR ShcKO mice are not entirely clear. Additional studies are needed to determine the influence of age and duration of CR on Shc-related changes in fatty acid oxidation and body composition.

## 5. Conclusions

In conclusion, the results of this study indicate that ShcKO and WT mice show similar changes in EE and body weight following 2 months of CR. However, Shc proteins do appear to influence the energetic response to very acute CR (3 days). In particular, knockdown of Shc proteins stimulates a rapid decrease in EE normalized for body weight in response to CR. Furthermore, Shc proteins may modulate CR-induced changes in whole animal substrate utilization in 3-month-old mice and may induce sustained increased lipid oxidation during periods of fasting in CR animals. Thus, Shc proteins should be considered as a possible factor contributing to energetic responses to very acute CR.

## Figures and Tables

**Table 1:** Cross-sectional comparison of organ weights and body composition in ShcKO and WT mice^1^.

	ShcKO			WT			*P* value		
	CON	CR	% ↓	CON	CR	% ↓	Geno	Treatment	Geno ∗ treatment
Body weight	26.19 ± 0.69^a^	20.85 ± 0.36^b^	20.3↓	27.26 ± 0.41^a^	22.17 ± 0.45^b^	18.6↓	0.020	<0.0001	0.807
Fat-free body Mass	22.46 ± 0.55^a∗^	18.34 ± 0.23^b^	18.8↓	24.23 ± 0.28^a∗^	19.72 ± 0.51^b^	18.6↓	0.0006	<0.0001	0.6384
Liver	1.17 ± 0.056^a^	0.96 ± 0.055^b^	17.9↓	1.32 ± 0.043^a^	1.08 ± 0.046^b^	18.1↓	0.013	<0.0001	0.834
Spleen	0.085 ± 0.0019^a^	0.039 ± 0.0014^b^	54.0↓	0.077 ± 0.0029^a^	0.038 ± 0.0025^b^	50.6↓	0.148	<0.0001	0.108
Kidneys	0.401 ± 0.011^a^	0.326 ± 0.015^b^	18.7↓	0.395 ± 0.011^a^	0.301 ± 0.0049^b^	23.7↓	0.164	<0.0001	0.383
Lungs	0.170 ± 0.011	0.145 ± 0.007	14.7↓	0.187 ± 0.0091	0.171 ± 0.0081	8.5↓	0.021	0.027	0.693
Heart	0.153 ± 0.0096	0.136 ± 0.0085	11.1↓	0.145 ± 0.0049	0.123 ± 0.0041	15.2↓	0.148	0.008	0.737
Brain	0.411 ± 0.011	0.390 ± 0.014	5.1↓	0.431 ± 0.0053	0.409 ± 0.0074	5.1↓	0.053	0.034	0.989
Crude organ Weight^2^	2.14 ± 0.064^a^	1.81 ± 0.047^b^	15.4↓	2.28 ± 0.048^a^	1.91 ± 0.046^b^	16.2↓	0.020	<0.0001	0.662

^1^Data are presented as means ± SEM; superscript letters that differ indicate differences between treatments (CON versus CR) within genotype, Bonferroni corrected *P* value < 0.05; ^2^crude organ weight is the sum of liver, spleen, kidney, lung, heart, and brain weights. *indicates difference between genotypes (ShcKO versus WT) within treatment, Bonferroni corrected *P* value < 0.05.

**Table 2:** Energy expenditure in response to ad libitum feeding, 3 days, and 2 months of calorie restriction^1^.

	ShcKO			WT			*P* value		
	Baseline	3-day CR	2-month CR	Baseline	3-day CR	2-month CR	Geno	Treatment	Geno ∗ treatment
24 hours									
EE (kJ/min/mouse)	0.0321 ± 5.37*E* − 4^a^	0.0263 ± 5.77*E* − 4^b^	0.0217 ± 5.18*E* − 4^c^	0.0314 ± 5.32*E* − 4^a^	0.0281 ± 1.06*E* − 3^b^	0.0222 ± 9.70*E* − 4^c^	0.589	<0.0001	0.163
EE_BW_ (kJ/min)^†^	0.0303 ± 7.14*E* − 4^a^	0.0271 ± 8.87*E* − 4^b^	0.0238 ± 7.93*E* − 4^c^	0.0293 ± 7.56*E* − 4^a^	0.0288 ± 7.56*E* − 4^a^	0.0224 ± 9.97*E* − 4^b^	0.823	<0.0001	0.135
Light									
EE (kJ/min/mouse)	0.0295 ± 5.60*E* − 4^a^	0.0205 ± 4.05*E* − 4^b^	0.0167 ± 7.31*E* − 4^c^	0.0289 ± 6.98*E* − 4^a^	0.0223 ± 1.19*E* − 3^b^	0.0171 ± 9.93*E* − 4^c^	0.493	<0.0001	0.261
EE_BW_ (kJ/min)^†^	0.0281 ± 6.30*E* − 4^a^	0.0213 ± 5.90*E* − 4^b^	0.0183 ± 8.80*E* − 4^b^	0.0271 ± 7.35*E* − 4^a^	0.0224 ± 5.63*E* − 4^a^	0.0176 ± 1.03*E* − 3^b^	0.891	<0.0001	0.305
Dark									
EE (kJ/min/mouse)	0.0348 ± 7.69*E* − 4^a^	0.0317 ± 8.97*E* − 4^a^	0.0264 ± 5.23*E* − 4^b^	0.0337 ± 6.36*E* − 4	0.0342 ± 8.38*E* − 4	0.0303 ± 3.02*E* − 3	0.247	0.001	0.082
EE_BW_ (kJ/min)^†^	0.0357 ± 9.13*E* − 4^a^	0.0313 ± 8.45*E* − 4^a^	0.0254 ± 6.74*E* − 4^b^	0.0348 ± 7.58*E* − 4	0.0338 ± 7.98*E* − 4	0.0301 ± 3.16*E* − 3	0.185	0.0003	0.079

Longitudinal analysis of energy expenditure in response to ad libitum feeding, 3 days, and 2 months of calorie restriction in ShcKO and WT mice.

BW: body weight; EE is expressed as EE kJ/min/mouse (kJ per min per mouse) and EE_BW _(kJ/min normalized by BW). ^1^Data is presented as means ± SEM unless otherwise indicated; superscript letters that differ indicate differences between treatment days within genotype, Bonferroni corrected *P* < 0.001; ^†^values are presented as least square mean ± SEM, adjusted for BW; treatment day refers to baseline, 3-day CR, and 2-month CR.

**Table 3:** Cross-sectional comparison of energy expenditure in control versus 2-month CR treatment.

	ShcKO		WT		*P* value		
	Baseline	2-month CR	Baseline	2-month CR	Geno	Treatment	Geno ∗ treatment
24 hours							
EE_FFM_ (kJ/min)^†^	0.0312 ± 7.90*E* − 4	0.0212 ± 1.38*E* − 3	0.0318 ± 1.12*E* − 3	0.0211 ± 1.69*E* − 3	0.797	<0.0001	0.654
EE_ORGAN _(kJ/min)^‡^	0.0309 ± 7.53*E* − 4	0.0223 ± 1.04*E* − 3	0.0309 ± 8.75*E* − 4	0.0216 ± 8.49*E* − 4	0.670	<0.0001	0.659
Light							
EE_FFM_ (kJ/min)^†^	0.0290 ± 7.99*E* − 4	0.0161 ± 1.40*E* − 3	0.0296 ± 1.13*E* − 3	0.0163 ± 1.40*E* − 3	0.725	<0.0001	0.756
EE_ORGAN _(kJ/min)^‡^	0.0287 ± 7.71*E* − 4	0.0172 ± 1.07*E* − 3	0.0288 ± 8.96*E* − 4	0.0167 ± 8.69*E* − 4	0.791	<0.0001	0.751
Dark							
EE_FFM_ (kJ/min)^†^	0.0333 ± 1.122*E* − 3	0.0260 ± 1.97*E* − 3	0.0339 ± 1.59*E* − 3	0.0258 ± 1.31*E* − 3	0.910	0.0021	0.683
EE_ORGAN _(kJ/min)^‡^	0.0331 ± 1.06*E* − 3	0.0272 ± 1.47*E* − 3	0.0329 ± 1.23*E* − 3	0.0263 ± 1.19*E* − 3	0.682	0.0006	0.693

Cross-sectional comparison of energy expenditure adjusted for FFM and ORGAN in control versus 2-month CR treatment ShcKO and WT mice.

FFM: fat-free mass; ORGAN: crude organ mass (sum of liver, kidney, heart, and brain mass); EE is expressed as EE_FFM, ORGAN_ (kJ/min normalized by FFM and ORGAN); ^†,‡^values are presented as least square mean ± SEM, adjusted for BW, FFM, and ORGAN, respectively. Treatment refers to baseline or 2-month CR.

## List of Abbreviations

(CR): Calorie restriction
(BW): Body weight
(EE): Energy expenditure
(EE_BW,FFM,ORGAN_): Energy expenditure normalized by BW, FFM, and ORGAN
(FFM): Fat-free mass
(ME): Metabolizable energy
(MEI): Metabolizable energy intake
(ORGAN): Crude organ mass
(RQ): Substrate utilization
(ShcKO): Shc knockdown
(TBW): Total body water
(WT): Wild-type.
